# A diabetes risk score for Qatar utilizing a novel mathematical modeling approach to identify individuals at high risk for diabetes

**DOI:** 10.1038/s41598-021-81385-3

**Published:** 2021-01-19

**Authors:** Susanne F. Awad, Soha R. Dargham, Amine A. Toumi, Elsy M. Dumit, Katie G. El-Nahas, Abdulla O. Al-Hamaq, Julia A. Critchley, Jaakko Tuomilehto, Mohamed H. J. Al-Thani, Laith J. Abu-Raddad

**Affiliations:** 1 Infectious Disease Epidemiology Group, Weill Cornell Medicine - Qatar, Cornell University, Qatar Foundation - Education City, Doha, Qatar; 2 World Health Organization Collaborating Centre for Disease Epidemiology Analytics On HIV/AIDS, Sexually Transmitted Infections, and Viral Hepatitis, Weill Cornell Medicine – Qatar, Cornell University, Qatar Foundation - Education City, P.O. Box 24144, Doha, Qatar; 3 Department of Population Health Sciences, Weill Cornell Medicine, Cornell University, New York, USA; 4 Public Health Department, Ministry of Public Health, Doha, Qatar; 5 Deloitte & Touche (M.E.), Doha, Qatar; 6 Qatar Diabetes Association, Doha, Qatar; 7 Population Health Research Institute, St George’s, University of London, London, UK; 8 Public Health Promotion Unit, Finnish Institute for Health and Welfare, Helsinki, Finland; 9 Department of Public Health, University of Helsinki, Helsinki, Finland; 10 Diabetes Research Group, King Abdulaziz University, Jeddah, Saudi Arabia

**Keywords:** Diabetes, Obesity, Risk factors, Applied mathematics, Statistics, Epidemiology

## Abstract

We developed a diabetes risk score using a novel analytical approach and tested its diagnostic performance to detect individuals at high risk of diabetes, by applying it to the Qatari population. A representative random sample of 5,000 Qataris selected at different time points was simulated using a diabetes mathematical model. Logistic regression was used to derive the score using age, sex, obesity, smoking, and physical inactivity as predictive variables. Performance diagnostics, validity, and potential yields of a diabetes testing program were evaluated. In 2020, the area under the curve (AUC) was 0.79 and sensitivity and specificity were 79.0% and 66.8%, respectively. Positive and negative predictive values (PPV and NPV) were 36.1% and 93.0%, with 42.0% of Qataris being at high diabetes risk. In 2030, projected AUC was 0.78 and sensitivity and specificity were 77.5% and 65.8%. PPV and NPV were 36.8% and 92.0%, with 43.0% of Qataris being at high diabetes risk. In 2050, AUC was 0.76 and sensitivity and specificity were 74.4% and 64.5%. PPV and NPV were 40.4% and 88.7%, with 45.0% of Qataris being at high diabetes risk. This model-based score demonstrated comparable performance to a data-derived score. The derived self-complete risk score provides an effective tool for initial diabetes screening, and for targeted lifestyle counselling and prevention programs.

## Introduction

Type 2 diabetes mellitus (T2DM) constitutes about 90% of all diabetes cases worldwide^1^. A “T2DM risk score” is an objective set of questions or measurements that can be used to assess the likelihood that an individual has undiagnosed T2DM, or a future risk of developing T2DM^2–4^, so that the subject can benefit from lifestyle advice and medical care to halt and potentially reverse progression to T2DM and its complications^4,5^. The utility of the risk score hinges on the benefits of earlier T2DM detection, given the severe complications that undiagnosed or poorly controlled T2DM can cause^6^. The International Diabetes Federation (IDF) recommends such scores as a population-based screening tool for T2DM^7^.

While different scores exist, most use a similar set of core variables, particularly age, a measure of anthropometry (body mass index [BMI] and/or waist circumference), family history of DM, history of gestational diabetes or other previous hyperglycemia, hypertension, etc^4^. Evidence shows moderate and variable diagnostic accuracy of such non-biochemical risk scores to detect undiagnosed T2DM or future risk of developing T2DM^4,8^. However, risk scores are still extremely useful in designing initial screening strategies and programs for T2DM, thereby reducing the need for more invasive, time consuming, and expensive blood glucose or glycated hemoglobin A1c (A1c) testing^8^. They are also likely to be a highly cost-effective means of screening for T2DM at the population level.

Few risk scores have been developed except for those in high-income settings, or validated in different populations—such scores may not perform well when validated elsewhere, compared with the initial derivation population^4,9^. Despite initiatives to provide effective risk scores for different populations, such as the IDF’s PREDICT-2 initiative^10^, few have been developed for populations in the Middle East and North Africa (MENA)^11–15^, the region harboring the highest T2DM prevalence worldwide, and projected to have the second largest proportional increase in the number of adults with T2DM by 2045, compared to other regions^16,17^. With limited availability of high-quality population-based data^18^, and in the context of rapidly increasing T2DM prevalence^1^, there is a critical need to devise risk scores that factor the dynamic epidemiology of T2DM and that can be implemented in wide-scale screening programs to identify undiagnosed T2DM and individuals at high risk.

Against this background, we sought to develop a novel analytical approach for deriving risk scores, using mathematical modeling that considers the evolving nature of the T2DM epidemic over time, even in the absence of repeated, high-quality, nationally representative population-based surveys of T2DM and its risk factors. Our approach is generic; thus, it can be applied to different populations and countries. Here, we apply it to the country of Qatar, as an illustrative example. We also estimate the testing yield of a T2DM testing program targeting different population strata in Qatar.

Qatar is a MENA country with one of the highest T2DM prevalence levels worldwide^1,19^. The 2012 Qatar STEPwise Survey reported a crude T2DM prevalence at 16.7% among adult Qatari nationals 18–64 years of age, of which one-third were unaware of their disease status^20^. Prevalence estimates of T2DM-related risk factors, specifically obesity, smoking, and physical inactivity, were also estimated at 41.4%, 16.4%, and 45.9%, respectively^20^.

## Methods

### Mathematical model

We previously developed a population-level, age-structured, dynamic mathematical model that projected up to 2050, the epidemiology of T2DM and its risk factors in the Qatari population, factoring in the dynamic interplay of demography and T2DM risk factors^19^. Briefly, the model stratified the Qatari population based on sex, age group, risk factor status, and T2DM status. The model disaggregated the population into males and females, and 20 five-year age bands (0–4, 5–9… 95–99 years old). It also incorporated two main disease states; not having T2DM and living with T2DM. It further incorporated major T2DM risk factors; obesity, smoking, and physical inactivity. “Overlaps” between these risk factors was accounted for by further stratifying the Qatari population into compartments with overlapping risk factors (such as being obese *and* a smoker at the same time)^19^. Obesity was defined as BMI ≥ 30 kg/m^2^_,_ smoking as those currently smoking tobacco daily, and physical inactivity as < 150 min of moderate activity and < 75 min of vigorous activity per week (i.e., < 600 metabolic equivalent-minutes per week)^20–22^.

The model was parameterized using representative epidemiological and demographic data for Qatar^19,20,23–25^, and was fitted to current country-specific epidemiological and demographic datasets to ensure that predictions of the model mimic the T2DM epidemiology of the Qatari population. Thus, sex- and age-specific prevalence data for T2DM, obesity, smoking, and physical inactivity for Qataris were obtained from the 2012 Qatar STEPwise survey^20^. Estimates of the relative risks of developing T2DM with respect to each key risk factor were obtained from large, high-quality prospective studies^23–25^. Through the fitting process, a set of unknown parameters were derived, generating the curves that best fitted to the data; hence, a future projection of the T2DM epidemic was possible. In other words, the mathematical model simulated T2DM and its key risk factors for the Qatari population for decades to come. Further details on model structure, its parametrization, model fitting, and assessment of robustness of model structure and predictions are found in Awad et al.^19^.

All modeling analyses were conducted using MATLAB 2019a^26^.

### Testing yield of a diabetes testing program

The testing yield, defined as the number of individuals needed to be tested in order to identify one T2DM case, was estimated from model projections for different sub-population strata, by sex, age, and risk factor status. The yield was derived as the inverse of the model prediction for the proportion of individuals living with T2DM in a given population stratum at each given time: 2020, 2030, and 2050.

### Development of the Qatari risk score

The model simulated the incidence and progression of T2DM and risk factors in the total Qatari population, that is, it generated a representation of the T2DM epidemic in the entire Qatari population in silico (i.e., through computer simulations). Subsequently, this in silico population was utilized by randomly sampling from it a total of 5,000 Qataris aged 15–79 years, i.e., the sampling frame was the entire simulated Qatari population. Sampling was implemented using a Monte Carlo sampling method. We used model classifications, outcomes, and projections, at four time points: 2012 (the year of the STEPwise survey, for validation), 2020, 2030, and 2050. Sex, age, obesity, smoking, and physical inactivity were the covariables incorporated for generating the risk score.

To derive a risk score for each time point, a multivariable logistic regression was performed by assigning each covariable a score based on the regression model’s β-coefficient, using established methodology^12^. For each covariable, the regression β-coefficient was multiplied by 10 and rounded to the nearest integer. The aggregate risk score for each individual in the sample was obtained by adding up the scores, thus ranging between 0 and 49. No interaction terms between the covariables were considered, to keep the score easy to use^27,28^.

### Assessment of the Qatari risk score performance

For each time point, performance of the risk score was evaluated by estimating the area under the receiver operating characteristic curve (AUC) and the probabilities of: a T2DM diagnosis given the individual has T2DM (*sensitivity*), a no-T2DM diagnosis given the individual does not have T2DM (*specificity*), of having T2DM given a T2DM diagnosis (*positive predictive value*; PPV), and of not having T2DM, given a no-T2DM diagnosis (*negative predictive value*; NPV).

The PPV was estimated as the proportion of individuals who were truly living with T2DM among those who were “identified” by the risk score as *having T2DM*. The NPV was estimated as the proportion of individuals who were truly not having T2DM among those who were “identified” by the risk score as *not having T2DM*.

The optimal cut-off score was chosen by maximizing the sum of the sensitivity and specificity. Consequently, the proportion of individuals who have a score greater than or equal to the cut-off was estimated, thus determining the proportion of individuals needing to be biochemically tested for T2DM.

Two sensitivity analyses were conducted for the 2020 sample in which the cut-off value was chosen based on increasing the specificity to 90%, to reduce false positives, thereby reducing the fraction of individuals needing to be biochemically tested for T2DM, and by increasing the sensitivity to 90%, to increase true positives, i.e., the proportion of individuals with T2DM detected by the risk score.

### Validation of the model-derived Qatari diabetes risk score

Using the above described methodology for deriving risk scores, we derived an independent diabetes risk score *directly* from the 2012 Qatar STEPwise Survey data^20^—that is *data-derived* risk score *not* using the model outcomes. To assess the validity of the *model-derived* Qatari diabetes risk score, we applied this risk score (for the year 2012) to the (empirical) sample of the 2012 Qatar STEPwise Survey^20^, and compared its performance to that of the *data-derived* risk score as applied to this same sample.

### Comparison with regional and international diabetes risk scores

Performance of the developed Qatari risk score was compared with validated regional and international risk scores that employ similar variables, especially obesity, which is critically important in the case of Qatar^19^. Regional risk scores were Omani^12^, Emirati^13^, and Saudi^14^, while international and widely-discussed scores^4^ were American^29^, Danish^30^, Dutch^28^, Finnish^27^, Taiwanese^31^, and Thai^32^. Each score was reanalyzed to evaluate its performance on the Qatari population in 2020 by including only the covariables in the Qatari sample (i.e., risk factors included in both the present study and other published risk-score studies). For each applied risk score, we recalculated the cut-off, maximizing the sum of sensitivity and specificity for the Qatari population.

All statistical analyses were conducted using IBM SPSS Statistics 25^33^.

## Results

### Characteristics of simulated samples

Of the 5000 Qataris in the simulated 2020 sample, prevalences of T2DM, obesity, smoking, and physical inactivity were 19.2%, 40.7%, 16.4%, and 49.3%, respectively (Table S1 of Supplementary Material [SM]). Similarly, in 2030, the prevalences were 20.4%, 43.8%, 16.6%, and 51.2%, respectively, and in 2050, they were 24.4%, 48.4%, 18.3%, and 57.0%, respectively (Table S1 of SM).

### Yields of a diabetes testing program

**Figure 1:**
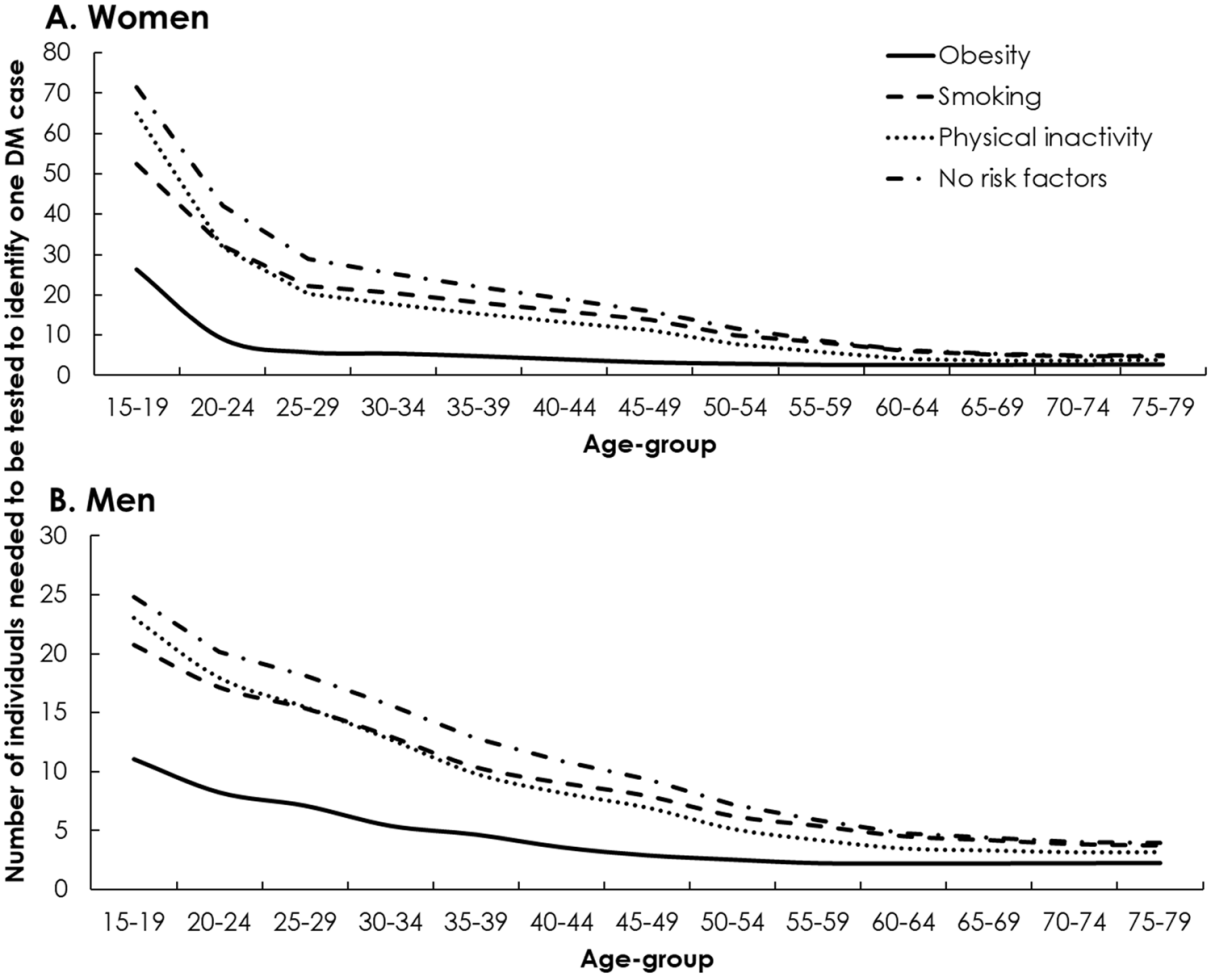
Yields of a screening program for diabetes mellitus (DM) targeting different subpopulation strata of (**A**) women and (**B**) men in 2020. The yield is defined as the number of individuals needed to be screened for DM to identify one DM case. The targeted subpopulations are stratified by age-group and obesity, smoking, and physical inactivity statuses.

Figure 1 and Table S2 of SM show the yields of a T2DM testing program, for each targeted subpopulation stratum. In 2020, numbers of obese, smoking, and physically inactive women that needed to be tested to identify one T2DM case ranged from 26.3, 52.4, and 64.8, respectively, for those 15–19 years old, to 2.7, 4.8, and 3.8, respectively, for those 75–79 years old (Fig. 1A). Similarly, numbers of obese, smoking, and physically inactive men that needed to be tested to identify one T2DM case ranged from 11.0, 20.8, and 23.0, respectively, for those 15–19 years old, to 2.3, 3.7, and 3.2, respectively, for those 75–79 years old (Fig. 1B). For individuals with none of these risk factors, the testing yield for women and men ranged from 67.6 and 23.3, respectively, for those 15–19 years old, to 4.9 and 3.9, respectively, for those 75–79 years old (Fig. 1). The yields in 2030 were relatively similar to those in 2020, while the yields in 2050 were superior to those in 2020 (Table S2).

### Univariable and multivariable logistic regression

Table 1 shows the univariable and multivariable logistic regression results for 2020, 2030, and 2050, and the specific risk score for each variable. All considered covariables were significantly associated with T2DM in univariable-level analyses and remained so at the multivariable level (Table 1).

**Table 1:** Multivariable logistic regression of risk factors for diabetes mellitus at three different time points: (A) 2020, (B) 2030, and (C) 2050.

	OR (95% CI)	aOR^#^ (95% CI)	β^$^	Risk score*
**(A) 2020**				
Age group				
15–19	Reference	–	–	0
20–24	2.24 (1.39–3.62)	1.80 (1.10–2.92)	0.59	6
25–29	4.01 (2.55–6.32)	2.77 (1.74–4.42)	1.02	10
30–34	4.63 (2.94–7.28)	2.63 (1.65–4.19)	0.97	10
35–39	7.65 (4.92–11.89)	4.14 (2.63–6.52)	1.42	14
40–44	9.16 (5.84–14.37)	4.68 (2.94–7.45)	1.54	15
45–49	11.82 (7.56–18.48)	6.00 (3.78–9.53)	1.79	18
50–54	13.08 (8.31–20.60)	7.54 (4.72–12.04)	2.02	20
55–59	20.48 (13.03–32.20)	11.64 (7.30–18.58)	2.46	25
60–64	18.00 (11.23–28.85)	11.77 (7.22–19.14)	2.46	25
65–69	15.40 (9.41–25.23)	10.92 (6.56–18.20)	2.39	24
70–74	15.75 (9.30–26.68)	13.75 (7.95–23.81)	2.62	26
75–79	13.57 (7.55–24.39)	11.58 (6.29–21.30)	2.45	24
Sex				
Women	Reference	–	–	0
Men	1.13 (0.98–1.3)	1.22 (1.03–1.46)	0.20	2
Obesity^¥^				
Non-obese	Reference	–	–	0
Obese	4.53 (3.89–5.28)	4.07 (3.44–4.82)	1.40	14
Smoking^#^				
Non-smoker	Reference	–	–	0
Smoker	1.5 (1.26–1.79)	1.36 (1.09–1.70)	0.31	3
Physical inactivity^€^				
Physically active	Reference	–	–	0
Physically inactive	2.26 (1.95–2.61)	1.70 (1.45–2.01)	0.53	5
Constant			− 4.10	
**(B) 2030**				
Age group				
15–19	Reference	–	–	0
20–24	1.88 (1.15–3.07)	1.52 (0.92–2.50)	0.42	4
25–29	3.25 (2.04–5.18)	2.20 (1.36–3.54)	0.79	8
30–34	4.24 (2.68–6.72)	2.41 (1.50–3.86)	0.88	9
35–39	5.54 (3.49–8.78)	2.87 (1.79–4.61)	1.05	11
40–44	7.43 (4.69–11.76)	3.79 (2.36–6.09)	1.33	13
45–49	10.87 (6.9–17.12)	5.73 (3.59–9.16)	1.75	17
50–54	12.83 (8.07–20.38)	7.13 (4.43–11.49)	1.96	20
55–59	12.97 (8.16–20.63)	7.35 (4.56–11.85)	2.00	20
60–64	15.85 (9.84–25.54)	10.72 (6.55–17.53)	2.37	24
65–69	13.47 (8.11–22.37)	9.81 (5.80–16.58)	2.28	23
70–74	13.11 (7.79–22.05)	10.54 (6.16–18.03)	2.36	24
75–79	8.26 (4.53–15.07)	7.54 (4.05–14.02)	2.02	20
Sex				
Women	Reference	–	–	0
Men	1.05 (0.92–1.21)	1.06 (0.90–1.26)	0.06	1
Obesity				
Non-obese	Reference	–	–	0
Obese	4.44 (3.82–5.16)	4.02 (3.40–4.74)	1.39	14
Smoking				
Non-smoker	Reference	–	–	0
Smoker	1.49 (1.25–1.77)	1.44 (1.16–1.77)	0.36	4
Physical inactivity				
Physically active	Reference	–	–	0
Physically inactive	1.85 (1.61–2.13)	1.52 (1.30–1.78)	0.42	4
Constant			− 3.95	
**(C) 2020**				
Age group				
15–19	Reference	–	–	0
20–24	1.38 (0.78–2.45)	1.05 (0.58–1.9)	0.05	1
25–29	2.30 (1.36–3.88)	1.37 (0.80–2.34)	0.31	3
30–34	3.85 (2.34–6.33)	2.01(1.20–3.36)	0.70	7
35–39	4.27 (2.61–6.99)	2.20 (1.32–3.66)	0.79	8
40–44	6.79 (4.19–11.01)	3.48 (2.11–5.74)	1.25	12
45–49	8.50 (5.26–13.75)	4.39 (2.67–7.21)	1.48	15
50–54	10.43 (6.44–16.88)	5.60 (3.41–9.21)	1.72	17
55–59	11.35 (7.01–18.38)	6.72 (4.09–11.04)	1.91	19
60–64	9.21 (5.62–15.11)	6.53 (3.92–10.88)	1.88	19
65–69	10.10 (6.12–16.65)	7.31 (4.36–12.26)	1.99	20
70–74	7.03 (4.13–11.94)	5.89 (3.40–10.22)	1.77	18
75–79	9.34 (5.38–16.21)	8.82 (4.97–15.66)	2.18	22
Sex				
Women	Reference	–	–	0
Men	1.20 (1.05–1.36)	1.35 (1.15–1.58)	0.29	3
Obesity				
Non-obese	Reference	–	–	0
Obese	4.14 (3.59–4.77)	4.39 (3.75–5.14)	1.48	15
Smoking				
Non-smoker	Reference	–	–	0
Smoker	1.28 (1.09–1.50)	1.24 (1.02–1.51)	0.22	2
Physical inactivity				
Physically active	Reference	–	–	0
Physically inactive	1.60 (1.40–1.83)	1.44 (1.24–1.67)	0.37	4
Constant			− 3.67	

The regression was used to derive the Qatari diabetes risk score.

^#^Odds ratios adjusted for age, sex, obesity, smoking, and physical inactivity.

^$^β-coefficients based on multivariable analysis.

*The maximum specific risk score for any given risk factor is 26 for 2020, 24 for 2030, and 22 for 2050.

^¥^Defined as body mass index ≥ 30 kg/m^2^^20^.

^#^Defined as those currently smoking tobacco daily^20^.

^€^Defined as < 150 min of moderate activity and < 75 min of vigorous activity per week (i.e., < 600 metabolic equivalent-minutes per week)^20^.

Overall, in the multivariable analysis, age and obesity were the strongest predictors for T2DM and contributed most to the risk score (Table 1). Individuals aged ≥ 55 were at substantially higher risk of T2DM compared to younger individuals. The specific risk score for age decreased with time, while the specific risk score for sex, obesity, smoking, and physical inactivity remained largely stable (Table 1).

For illustration, the 2020 Qatari diabetes risk score was expressed using the formula illustrated in Box 1.

**Box 1:**
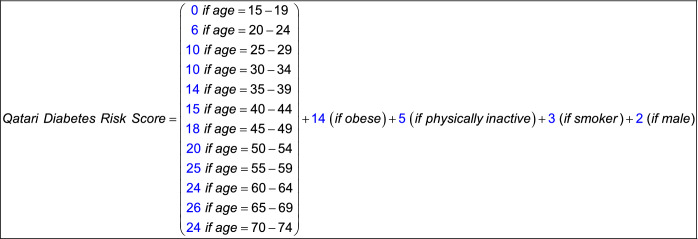
Formula for the Qatari diabetes risk score for 2020.

### Performance of the Qatari diabetes risk score

**Figure 2:**
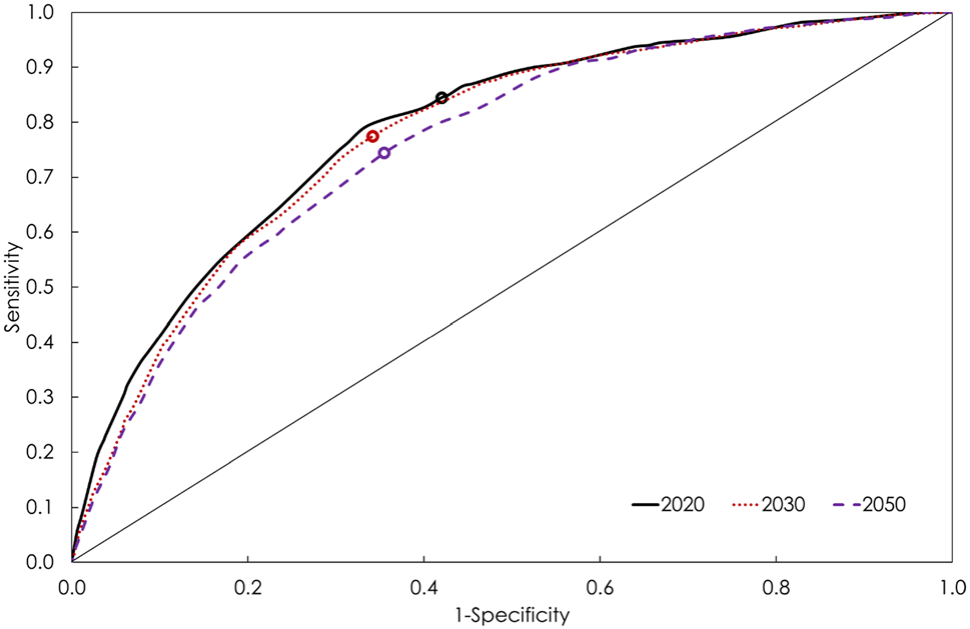
Receiver operating characteristic curves showing the performance of the Qatari diabetes risk score in diagnosing diabetes mellitus among Qataris at three time points: 2020, 2030, and 2050. The area under the curve (AUC) was 0.79 for the 2020 risk score, 0.78, for the 2030 risk score, and 0.76 for the 2050 risk score.

In 2020, the AUC was 0.79 (95% confidence interval [CI] 0.77–0.80; Table 2 and Fig. 2). The optimal combination of sensitivity of 79.0% (95% CI 76.3–81.4%) and specificity of 66.8% (95% CI 65.3–68.2%) was obtained at a score cut-off value of 26.5 (Table 2). PPV and NPV were 36.1% (95% CI 34.1–38.2%) and 93.0% (95% CI 92.1–93.9%), respectively. With a cut-off value of 26.5, 42.0% (95% CI 40.6–43.4%) of Qataris aged 15–79 years old were at high risk of having undiagnosed T2DM (that is a risk score value above or equal the cut-off value), and therefore recommended for glycemia testing (Table 2).

**Table 2:** Performance of the Qatari diabetes risk score at three different time points: 2020, 2030, and 2050.

Year	AUC (95% CI)	Sensitivity (%; 95% CI)	Specificity (%; 95% CI)	PPV (%; 95% CI)	NPV (%; 95% CI)	Risk score cut-off^€^	Proportion needed testing^£^ (%; 95% CI)
2020	0.79 (0.77–0.80)	79.0 (76.3–81.4)	66.8 (65.3–68.2)	36.1 (34.1–38.2)	93.0 (92.1–93.9)	26.5	42.0 (40.6–43.4)
2030	0.78 (0.76–0.79)	77.5 (74.9–80.0)	65.8 (64.3–67.3)	36.8 (34.8–38.8)	92.0 (90.9–92.9)	24.5	43.0 (41.6–44.4)
2050	0.76 (0.75–0.78)	74.4 (71.9–76.8)	64.5 (62.9–66.0)	40.4 (38.4–42.4)	88.7 (87.4–89.8)	25.5	45.0 (43.6–46.4)

*AUC* area under the curve; *CI* confidence interval; *PPV* positive predictive value; *NPV* negative predictive value.

^€^The risk score cut-off was chosen based on the maximum sum of sensitivity and specificity.

^£^Proportion of individuals who had a risk score greater or equal to the cut-off value.

In 2030, the AUC was 0.78 (95% CI 0.76–0.79; Table 2 and Fig. 2). The optimal combination of sensitivity of 77.5% (95% CI 74.9–80.0%) and specificity of 65.8% (95% CI 64.3–67.3%) was obtained at a score cut-off value of 24.5 (Table 2). PPV and NPV were 36.8% (95% CI 34.8–38.8%) and 92.0% (95% CI 90.9–92.9%), respectively. With a cut-off of 24.5, 43.0% (95% CI 41.6–44.4%) of Qataris aged 15–79 years old were at high risk of having undiagnosed T2DM (Table 2).

In 2050, the AUC was 0.76 (95% CI 0.75–0.78; Table 2 and Fig. 2). The optimal combination of sensitivity of 74.4% (95% CI 71.9–76.8%) and specificity of 64.5% (95% CI 62.9–66.0%) was obtained at a cut-off of 25.5 (Table 2). PPV and NPV were 40.4% (95% CI 38.4–42.4%) and 88.7% (95% CI 87.4–89.8%), respectively. With a cut-off of 25.5, 45.0% (95% CI 43.6–46.4%) of Qataris aged 15–79 years old were at high risk of having undiagnosed T2DM (Table 2).

In the sensitivity analysis in which the cut-off value was 34.5, a value chosen to achieve a specificity of 90%, 15.8% (95% CI 14.8–16.9%) of Qataris aged 15–79 years old were at high risk of having undiagnosed T2DM in 2020, and would therefore be recommended for glycemia testing. By maximizing the specificity, 59.1% of T2DM cases would be missed.

In the sensitivity analysis, in which the cut-off was 18.5, a value chosen to achieve a sensitivity of 90%, 59.7% (95% CI 58.3–61.0%) of Qataris aged 15–79 years old were at high risk of having undiagnosed T2DM in 2020, and would therefore be recommended for glycemia testing. By maximizing sensitivity, only 10.0% of T2DM cases would be missed.

### Validation of the model-derived Qatari diabetes risk score

Table S3 shows the data-derived risk score using the 2012 Qatar STEPwise Survey data. Table S4 shows also the 2012 model-derived risk score, as derived using the model outcomes.

Table S5 shows performance of these two risk scores when both are applied to the 2012 STEPwise survey sample. For the model-derived risk score, the AUC was 0.69 (95% CI 0.66–0.72), similar to the AUC of the data-derived risk score of 0.70 (95% CI 0.68–0.73). Diagnostic performance was affirmed similar for both of these scores.

### Comparison with regional and international diabetes risk scores

Table 3 shows performance of regional (Emirati, Omani, and Saudi) and international (American, Danish, Dutch, Finnish, Taiwanese, and Thai) risk scores as applied to the 2020 Qatari sample. For all risk scores, the AUC ranged between 0.71 and 0.77; lower than the AUC of the Qatari risk score (0.79). Of the regional risk scores, the Emirati score had the largest AUC at 0.76 (95% CI 0.74–0.78) with a sensitivity of 62.5% (95% CI 59.4–65.5%) and a specificity of 77.4% (95% CI 76.1–78.7%). Of the international risk scores, the Danish score had the largest AUC at 0.77 (95% CI 0.76–0.79) with a sensitivity of 76.1% (95% CI 73.3–78.7%) and a specificity of 66.7% (95% CI 65.3–68.2%).

**Table 3:** Performance of three regional and four international diabetes risk scores in predicting diabetes mellitus among Qataris in 2020.

Risk score	AUC (95% CI)	Sensitivity (%; 95% CI)	Specificity (%; 95% CI)	Risk score cut-off*
Qatari	0.79 (0.77–0.80)	79.0 (76.3–81.4)	66.8 (65.3–68.2)	> 26.50
**Regional scores**				
Emirati	0.76 (0.74–0.78)	62.5 (59.4–65.5)	77.4 (76.1–78.7)	> 8.50
Omani	0.75 (0.73–0.77)	56.7 (53.6–59.8)	79.6 (78.3–80.8)	> 8.00
Saudi	0.71 (0.70–0.73)	70.8 (67.8–73.6)	64.0 (62.5–65.5)	> 4.00
**International scores**				
American	0.74 (0.72–0.76)	71.2 (68.2–74.0)	66.1 (64.6–67.5)	≥ 2.25
Danish	0.77 (0.76–0.79)	76.1 (73.3–78.7)	66.7 (65.3–68.2)	≥ 20.00
Dutch	0.71 (0.69–0.72)	72.0 (69.1–74.8)	61.5 (60.0–63.0)	≥ 10.50
Finnish	0.76 (0.74–0.78)	84.8 (82.4–86.9)	55.5 (53.9–57.0)	≥ 2.50
Taiwanese	0.76 (0.75–0.79)	84.3 (81.8–86.5)	56.8 (55.3–58.4)	≥ 1.03
Thai	0.74 (0.72–0.76)	80.3 (77.7–82.7)	59.7 (58.2–61.2)	> 3.50

*AUC* area under the curve; *CI* confidence interval.

*For each risk score, the cut-off was recalculated to maximize sum of sensitivity and specificity for the Qatari sample.

The Finnish, Taiwanese, and Thai risk scores showed very similar performance, and had the highest sensitivities at 84.8% (95% CI 82.4–86.9%), 84.3% (95% CI 81.8–86.5%), and 80.3% (95% CI 77.7–82.7%), respectively; but (predictably) had the lowest specificities at 55.5% (95% CI 53.9–57.0%), 56.8% (95% CI 55.3–58.4%), and 59.7% (95% CI 58.2–61.2%), respectively. Of all risk scores, the Omani risk score showed the lowest sensitivity at 56.7% (95% CI 53.6–59.8%), but the highest specificity at 79.6% (95% CI 78.3–80.8%).

## Discussion

In addition to existing published methodologies for deriving diabetes risk scores, which are mostly based on logistic regression analyses of cross-sectional or prospective data^2–4^, we demonstrated a new methodology with broad utility and application. There are two major advantages to this new approach compared to existing methods. First, it can be applied to countries with limited or insufficient nationally-representative population-based survey data. Second, it dynamically factors the temporal evolution of T2DM epidemics and T2DM risk factors; thus, it can provide risk scores at variable time points in the future.

The presented approach is especially suited for countries with inconsistent, or apparently conflicting survey data as well as in countries where data are limited or sparse (such as in MENA, Africa, or other low-income countries), but where conducting T2DM modeling informed by a global understanding of T2DM epidemiology is possible. Many countries may have different population-based surveys, but the data are difficult to reconcile due to variations in survey quality, time, design, geographic coverage, and methods to ascertain T2DM and risk factors, in addition to inconsistent definitions of outcomes and differences in response rates among others^18,34–36^. By using the introduced modeling approach, model fitting will ensure that the best fit to the data is reached, factoring all existing survey data, adjustments/corrections to these data, and weights for the level of confidence in data from each survey, irrespective of discrepancies and limitations in available data.

Here we applied this methodology to Qatar, one of the most T2DM-burdened nations worldwide. T2DM prevalence in this nation was projected to reach 24.0% by 2050, with a relative increase of 43% between 2012 and 2050^19^. Close to one-third of national health expenditure in 2050 was predicted to be spent on tackling T2DM and its complications^19^. These figures highlight the urgency of cost-effective interventions for early detection of undiagnosed T2DM cases, such as the use of risk scores. This approach might also be useful as a tool for screening campaigns and programs, and to disseminate awareness and increase knowledge about T2DM and its risk factors. Our model-derived risk score, though simple to implement and non-invasive, demonstrated adequately high diagnostic accuracy with a PPV of 36% and a NPV of 93% in 2020 (Table 2). Importantly, its application to empirical survey data demonstrated a performance similar to that of a data-derived risk score (Table S5) affirming the reliability of this approach.

Results of this model-derived score showed that a large proportion of the adult Qatari population (> 42%) has a score above or equal to the cut-off value of the score; hence, the need to be tested for glycaemia on regular basis (Table 2). The model-derived risk score indicated that virtually any Qatari older than 55 years of age, or any Qatari living with obesity and older than 35 years of age, is at high risk of having undiagnosed T2DM (Table 1), and should be regularly tested for it. Similarly, recent results from developing a risk score in Jeddah, Saudi Arabia showed that everyone aged 50 years or older should be tested for glycaemia, since more than half of people in this age group have it^15^. The presented results also demonstrated large variations in the yields of T2DM testing by sex, age, and T2DM risk factors as well as over time (Fig. 1 and Table S2). The best yields of T2DM testing were attained for those older than 50 years of age, or those living with obesity, where generally well below 10 tests are needed to diagnose an individual living with T2DM.

Findings of the model-derived score indicated that despite some variation, the structure and coefficients of the risk score were only minimally variable over time (Table 1). The same was true for the proportion of Qataris that needed to be regularly tested for T2DM, which only varied between 42% in 2020 and 45% in 2050 (Table 2). Though the model-derived Qatari diabetes risk score demonstrated superior performance on the Qatari population compared to that of other regional and international risk scores (Table 3), the other risk scores still showed good diagnostic accuracy, suggesting the universality of some aspects of the global T2DM epidemic—in particular the effects of age, ageing cohorts, and obesity.

This study has some limitations. Even though the risk score was derived from a sample generated directly from the model outcomes, it did not have a perfect performance compared to the model outcomes (Table 2 and Fig. 2). By design, a risk score has to be simple in structure for ease of use; therefore, it cannot fully represent the rich modelled T2DM dynamics, such as overlap and interactions of the different T2DM risk factors^19^. We developed the risk score from model-simulated, population-based samples, akin to how risk scores are derived using samples recruited through cross-sectional, population-based surveys^12–14,28,30,32^, thereby yielding an accessible risk score that can be used broadly, both in health facilities and by the general population. The approach was also validated by comparing the model-derived risk score to that of a data-derived risk score (Table S5). Yet, the 2012 Qatar STEPwise Survey data used to validate the score were also part of the input data used to calibrate the model. Preferably, validation of the score should be based on fully independent data such as those of the next planned STEPwise Survey.

Limitations in the input data have affected the number of factors that could be included in the risk score, as well as its application to non-Qataris residing in Qatar. However, given that diabetes risk scores developed in other populations also showed good accuracy in detecting T2DM (Table 3), this provides assurance that the risk score developed here could be of utility to non-Qatari residents. Variables originally included in the mathematical model also affected the factors that could be included in the risk score. For instance, the risk score did not include family history of diabetes as this factor was not part of the original mathematical model. However, as more population-based data become available, there will be opportunities to expand the mathematical model and to refine this score by including other factors such as family history among others.

The score cut-off value was chosen by maximizing the sum of sensitivity and specificity, but other approaches could have been used, as required by any specific program, such as the need to maximize sensitivity or specificity, presented here in sensitivity analyses. Clearly, maximizing specificity will always be more efficient, but has a “cost” of missing many people with undetected T2DM. We compared our risk score with some regional and international scores, but we could not compare with other scores due to insufficient overlap with the variables used in our risk score^11,37–39^. Finally, this novel method of deriving risk scores remains to be further tested and validated by applying it to different populations and learning from these experiences.

In conclusion, a diabetes risk score for Qataris, based on a set of non-invasive and easy-to-capture variables, was derived using an innovative approach of broad utility and application, and it can account for temporal variation in T2DM epidemiology. The model-derived score demonstrated diagnostic accuracy and comparable performance to that of a data-derived score. It also identified population strata that should be prioritized for testing for glycaemia and preventive interventions. With the above findings, the developed self-complete score can be easily implemented as part of awareness campaigns and initial screening programs to determine the need for invasive biochemical testing, or to prioritize individuals for lifestyle counselling and T2DM prevention programs.

## Supplementary Information

Supplementary Information.

## Data Availability

MATLAB codes for the model can be obtained from the authors.
