# Supplementary material for: A diabetes risk score for Qatar utilizing a novel mathematical modeling approach to identify individuals at high risk for diabetes
Source: Sci Rep. 2021 Jan 19;11:1811. doi: 10.1038/s41598-021-81385-3 (PMC7815783; doi:10.1038/s41598-021-81385-3)
Supplement: Supplementary file 1 — Supplementary Information. [file 41598_2021_81385_MOESM1_ESM.docx]

**A diabetes risk score for Qatar utilizing a novel mathematical modeling approach to identify individuals at high risk for diabetes**

**Supplementary Material**

Susanne F. Awad,^1,2,3^ Soha R. Dargham,^1,2^ Amine A. Toumi,^4^ Elsy M. Dumit,^5^ Katie G. El-Nahas,^6^ Abdulla O. Al-Hamaq,^6^ Julia A. Critchley,^7^ Jaakko Tuomilehto,^8,9,10^ Mohammed H. J. Al-Thani,^4^ and Laith J. Abu-Raddad^1,2,3^

^1^Infectious Disease Epidemiology Group, Weill Cornell Medicine - Qatar, Cornell University, Qatar Foundation - Education City, Doha, Qatar

^2^World Health Organization Collaborating Centre for Disease Epidemiology Analytics on HIV/AIDS, Sexually Transmitted Infections, and Viral Hepatitis, Weill Cornell Medicine – Qatar, Cornell University, Qatar Foundation – Education City, Doha, Qatar

^3^Department of Population Health Sciences, Weill Cornell Medicine, Cornell University, New York, USA

^4^Public Health Department, Ministry of Public Health, Doha, Qatar

^5^Deloitte & Touche (M.E.), Doha, Qatar

^6^Qatar Diabetes Association, Doha, Qatar

^7^Population Health Research Institute, St George’s, University of London, London, UK

^8^Public Health Promotion Unit, Finnish Institute for Health and Welfare, Helsinki, Finland

^9^Department of Public Health, University of Helsinki, Helsinki, Finland

^10^Diabetes Research Group, King Abdulaziz University, Jeddah, Saudi Arabia

**Table S1.** Characteristics of the Qatari sample at three time points: 2020, 2030, and 2050.

|  |  | **2020** | **2030** | **2050** |
| --- | --- | --- | --- | --- |
|  |  | **N (%)** | **N (%)** | **N (%)** |
| **Sample size** |  | 5,000 | 5,000 | 5,000 |
| **Age group**  **(years)** | 15-19 | 785 (15.7) | 597 (11.9) | 391 (7.8) |
|  | 20-24 | 689 (13.8) | 684 (13.7) | 413 (8.3) |
|  | 25-29 | 592 (11.8) | 619 (12.4) | 468 (9.4) |
|  | 30-34 | 544 (10.9) | 569 (11.4) | 480 (9.6) |
|  | 35-39 | 481 (9.6) | 472 (9.4) | 487 (9.7) |
|  | 40-44 | 374 (7.5) | 408 (8.2) | 471 (9.4) |
|  | 45-49 | 351 (7.0) | 382 (7.6) | 464 (9.3) |
|  | 50-54 | 302 (6.0) | 309 (6.2) | 425 (8.5) |
|  | 55-59 | 275 (5.5) | 304 (6.1) | 411 (8.2) |
|  | 60-64 | 215 (4.3) | 237 (4.7) | 332 (6.6) |
|  | 65-69 | 175 (3.5) | 170 (3.4) | 291 (5.8) |
|  | 70-74 | 128 (2.6) | 151 (3.0) | 214 (4.3) |
|  | 75-79 | 89 (1.8) | 98 (1.96) | 153 (3.1) |
| **Diabetes mellitus** | No | 4,039 (80.8) | 3,980 (79.6) | 3,779 (75.6) |
|  | Yes | 961 (19.2) | 1,020 (20.4) | 1,221 (24.4) |
| **Sex** | Women | 2,340 (46.8) | 2,365 (47.3) | 2,268 (45.4) |
|  | Men | 2,660 (53.2) | 2,635 (52.7) | 2,732 (54.6) |
| **Obesity**^¥^ | No | 2,964 (59.3) | 2,808 (56.2) | 2,578 (51.6) |
|  | Yes | 2,036 (40.7) | 2,192 (43.8) | 2,422 (48.4) |
| **Smoking^#^** | No | 4,178 (83.6) | 4,172 (83.4) | 4,087 (81.7) |
|  | Yes | 822 (16.4) | 828 (16.6) | 913 (18.3) |
| **Physical Inactivity^€^** | No | 2,534 (50.7) | 2,438 (48.8) | 2,150 (43.0) |
|  | Yes | 2,466 (49.3) | 2,562 (51.2) | 2,850 (57.0) |

^¥^Defined as body mass index ≥ 30 kg/m^2^.^1^

^#^Defined as those currently smoking tobacco daily.^1^

^€^Defined as <600 metabolic equivalent-minutes per week (i.e., <150 minutes per week of brisk walking or <75 minutes per week of running).^1^

**Table S2.** Number of individuals needed to be biologically screened to identify one type 2diabetes mellitus (T2DM) case in a screening program, stratified by sex, age, and T2DM-related risk-factors of obesity, smoking, and physical inactivity, for three time periods: 2020, 2030, and 2050.

**A. Women**

| Age | Obese | | | Smoker | | | Physically Inactive | | | Obese Smoker | | | Obese Physically Inactive | | | Smoker Physically Inactive | | | Obese, Smoker, & Physically Inactive | | |
| --- | --- | --- | --- | --- | --- | --- | --- | --- | --- | --- | --- | --- | --- | --- | --- | --- | --- | --- | --- | --- | --- |
|  | **2020** | **2030** | **2050** | **2020** | **2030** | **2050** | **2020** | **2030** | **2050** | **2020** | **2030** | **2050** | **2020** | **2030** | **2050** | **2020** | **2030** | **2050** | **2020** | **2030** | **2050** |
| 15-19 | 26.3 | 23.7 | 22.5 | 52.4 | 46.9 | 43.9 | 64.8 | 58.9 | 53.7 | 20.9 | 19.4 | 17.4 | 20.1 | 18.8 | 16.5 | 46.5 | 17.8 | 15.7 | 15.7 | 15.1 | 13.0 |
| 20-24 | 9.1 | 8.5 | 7.7 | 32.1 | 29.7 | 26.8 | 31.6 | 29.6 | 26.0 | 6.8 | 6.5 | 5.7 | 5.8 | 5.6 | 4.9 | 23.5 | 13.7 | 11.9 | 4.5 | 4.4 | 3.8 |
| 25-29 | 5.7 | 5.5 | 4.8 | 22.3 | 21.2 | 18.7 | 20.3 | 19.4 | 16.8 | 4.4 | 4.3 | 3.8 | 3.7 | 3.6 | 3.2 | 15.5 | 12.1 | 10.5 | 2.9 | 2.9 | 2.6 |
| 30-34 | 5.4 | 5.3 | 4.6 | 20.4 | 19.8 | 17.2 | 17.6 | 17.2 | 14.9 | 4.4 | 4.3 | 3.8 | 3.5 | 3.5 | 3.1 | 14.1 | 10.1 | 8.8 | 2.9 | 2.9 | 2.6 |
| 35-39 | 4.8 | 4.7 | 4.2 | 18.2 | 17.9 | 15.6 | 15.3 | 15.1 | 13.3 | 3.9 | 3.9 | 3.5 | 3.1 | 3.1 | 2.9 | 12.6 | 7.7 | 6.7 | 2.7 | 2.7 | 2.5 |
| 40-44 | 4.0 | 4.0 | 3.6 | 16.0 | 15.8 | 14.1 | 13.1 | 13.1 | 11.7 | 3.4 | 3.4 | 3.1 | 2.7 | 2.7 | 2.5 | 11.1 | 6.7 | 6.1 | 2.3 | 2.4 | 2.2 |
| 45-49 | 3.2 | 3.2 | 3.0 | 13.8 | 13.7 | 12.5 | 11.1 | 11.1 | 10.2 | 2.8 | 2.8 | 2.7 | 2.3 | 2.3 | 2.2 | 9.6 | 5.9 | 5.5 | 2.0 | 2.0 | 2.0 |
| 50-54 | 2.9 | 2.9 | 2.8 | 10.0 | 10.0 | 9.2 | 7.7 | 7.7 | 7.2 | 2.6 | 2.6 | 2.5 | 2.1 | 2.1 | 2.0 | 6.7 | 4.5 | 4.3 | 1.9 | 1.9 | 1.9 |
| 55-59 | 2.6 | 2.6 | 2.5 | 8.2 | 8.2 | 7.6 | 5.8 | 5.8 | 5.5 | 2.4 | 2.4 | 2.3 | 1.9 | 1.9 | 1.9 | 5.5 | 3.9 | 3.8 | 1.8 | 1.8 | 1.8 |
| 60-64 | 2.6 | 2.6 | 2.5 | 6.1 | 6.1 | 5.8 | 4.2 | 4.2 | 4.1 | 2.4 | 2.4 | 2.3 | 1.9 | 1.9 | 1.9 | 4.1 | 3.3 | 3.2 | 1.8 | 1.8 | 1.8 |
| 65-69 | 2.6 | 2.6 | 2.6 | 5.2 | 5.2 | 5.0 | 3.8 | 3.7 | 3.7 | 2.4 | 2.4 | 2.4 | 2.0 | 2.0 | 2.0 | 3.6 | 3.2 | 3.1 | 1.8 | 1.8 | 1.8 |
| 70-74 | 2.7 | 2.7 | 2.6 | 4.8 | 4.8 | 4.7 | 3.7 | 3.6 | 3.6 | 2.6 | 2.6 | 2.6 | 2.1 | 2.1 | 2.1 | 3.6 | 3.0 | 2.9 | 2.1 | 2.1 | 2.0 |
| 75-79 | 2.7 | 2.7 | 2.7 | 4.8 | 4.8 | 4.6 | 3.8 | 3.8 | 3.7 | 2.7 | 2.6 | 2.6 | 2.2 | 2.2 | 2.2 | 3.7 | 3.0 | 3.0 | 2.2 | 2.2 | 2.1 |

**B. Men**

| Age | Obese | | | Smoker | | | Physically Inactive | | | Obese Smoker | | | Obese Physically Inactive | | | Smoker Physically Inactive | | | Obese, Smoker, & Physically Inactive | | |
| --- | --- | --- | --- | --- | --- | --- | --- | --- | --- | --- | --- | --- | --- | --- | --- | --- | --- | --- | --- | --- | --- |
|  | **2020** | **2030** | **2050** | **2020** | **2030** | **2050** | **2020** | **2030** | **2050** | **2020** | **2030** | **2050** | **2020** | **2030** | **2050** | **2020** | **2030** | **2050** | **2020** | **2030** | **2050** |
| 15-19 | 11.0 | 10.0 | 9.3 | 20.8 | 18.7 | 17.5 | 23.0 | 21.0 | 19.3 | 9.5 | 8.9 | 7.9 | 8.6 | 8.1 | 7.1 | 19.0 | 43.2 | 37.5 | 7.1 | 6.8 | 5.9 |
| 20-24 | 8.2 | 7.7 | 6.8 | 17.1 | 15.9 | 14.2 | 17.9 | 16.8 | 14.8 | 6.2 | 5.9 | 5.2 | 5.8 | 5.6 | 4.9 | 14.3 | 22.5 | 19.4 | 4.4 | 4.3 | 3.7 |
| 25-29 | 7.1 | 6.8 | 5.9 | 15.4 | 14.6 | 12.7 | 15.5 | 14.8 | 12.8 | 5.4 | 5.3 | 4.6 | 5.0 | 4.9 | 4.2 | 12.5 | 15.1 | 13.1 | 3.8 | 3.8 | 3.3 |
| 30-34 | 5.4 | 5.3 | 4.6 | 13.0 | 12.6 | 10.9 | 12.7 | 12.4 | 10.7 | 4.1 | 4.1 | 3.6 | 3.8 | 3.7 | 3.3 | 10.2 | 13.9 | 12.2 | 2.9 | 2.9 | 2.7 |
| 35-39 | 4.7 | 4.6 | 4.0 | 10.4 | 10.2 | 8.7 | 9.8 | 9.7 | 8.2 | 3.7 | 3.6 | 3.3 | 3.3 | 3.3 | 3.0 | 7.8 | 12.5 | 11.2 | 2.6 | 2.6 | 2.5 |
| 40-44 | 3.6 | 3.6 | 3.2 | 9.1 | 9.0 | 7.9 | 8.2 | 8.1 | 7.2 | 3.0 | 2.9 | 2.7 | 2.6 | 2.6 | 2.4 | 6.7 | 11.0 | 10.1 | 2.2 | 2.2 | 2.1 |
| 45-49 | 2.9 | 2.9 | 2.7 | 8.0 | 7.9 | 7.1 | 6.9 | 6.9 | 6.3 | 2.5 | 2.5 | 2.4 | 2.1 | 2.1 | 2.1 | 5.9 | 9.6 | 9.0 | 1.9 | 1.9 | 1.8 |
| 50-54 | 2.5 | 2.5 | 2.4 | 6.3 | 6.2 | 5.7 | 5.1 | 5.1 | 4.8 | 2.2 | 2.2 | 2.1 | 1.9 | 1.9 | 1.9 | 4.5 | 6.7 | 6.4 | 1.7 | 1.7 | 1.7 |
| 55-59 | 2.2 | 2.2 | 2.2 | 5.4 | 5.4 | 5.1 | 4.2 | 4.2 | 4.0 | 2.0 | 2.0 | 2.0 | 1.7 | 1.7 | 1.7 | 3.9 | 5.5 | 5.3 | 1.6 | 1.6 | 1.6 |
| 60-64 | 2.2 | 2.2 | 2.2 | 4.5 | 4.5 | 4.3 | 3.5 | 3.5 | 3.4 | 2.0 | 2.0 | 2.0 | 1.7 | 1.7 | 1.7 | 3.3 | 4.1 | 4.0 | 1.6 | 1.6 | 1.6 |
| 65-69 | 2.2 | 2.2 | 2.2 | 4.2 | 4.2 | 4.1 | 3.3 | 3.3 | 3.2 | 2.1 | 2.1 | 2.0 | 1.8 | 1.8 | 1.8 | 3.2 | 3.6 | 3.5 | 1.7 | 1.7 | 1.7 |
| 70-74 | 2.2 | 2.2 | 2.2 | 3.9 | 3.9 | 3.8 | 3.1 | 3.1 | 3.1 | 2.1 | 2.1 | 2.1 | 1.8 | 1.8 | 1.8 | 3.0 | 3.6 | 3.5 | 1.7 | 1.7 | 1.7 |
| 75-79 | 2.3 | 2.3 | 2.2 | 3.7 | 3.7 | 3.7 | 3.2 | 3.1 | 3.1 | 2.1 | 2.1 | 2.1 | 1.9 | 1.9 | 1.9 | 3.1 | 3.7 | 3.6 | 1.8 | 1.8 | 1.8 |

**Table S3.** Multivariable logistic regression of risk factors for diabetes mellitus on the 2012 Qatar STEPwise Survey data^1^ to formulate the *data-derived* (empirical) risk score.

|  |  | **OR (95% CI)** | **aOR (95% CI)** | **β** | **Risk score*** |
| --- | --- | --- | --- | --- | --- |
| **Age group** | **15-19** | Reference | - | - | **0** |
|  | **20-24** | 0.69 (0.25-1.88) | 0.67 (0.24-1.83) | -0.40 | **-4** |
|  | **25-29** | 0.99 (0.37-2.68) | 0.95 (0.35-2.58) | -0.05 | **-1** |
|  | **30-34** | 1.34 (0.54-3.28) | 1.19 (0.48-2.94) | 0.18 | **2** |
|  | **35-39** | 1.33 (0.55-3.21) | 1.18 (0.48-2.87) | 0.17 | **2** |
|  | **40-44** | 3.02 (1.31-6.96) | 2.56 (1.1-5.95) | 0.94 | **9** |
|  | **45-49** | 4.07 (1.74-9.49) | 3.38 (1.43-7.96) | 1.22 | **12** |
|  | **50-54** | 5.95 (2.52-14.01) | 4.98 (2.09-11.86) | 1.61 | **16** |
|  | **55-59** | 4.91 (1.99-12.12) | 4.06 (1.63-10.12) | 1.40 | **14** |
|  | **60-64** | 8.56 (3.49-20.99) | 7.24 (2.93-17.89) | 1.98 | **20** |
|  | **65-69** | - | - | - | **-** |
|  | **70-74** | - | - | - | **-** |
|  | **75-79** | - | - | - | **-** |
| **Sex** | **Women** | Reference | - | - | **0** |
|  | **Men** | 1.16 (0.88-1.52) | 1.2 (0.87-1.65) | 0.18 | **2** |
| **Obesity**^¥^ | **Non-obese** | Reference | - | - | **0** |
|  | **Obese** | 1.85 (1.41-2.41) | 1.55 (1.16-2.06) | 0.44 | **4** |
| **Smoking**^#^ | **Non-smoker** | Reference | - | - | **0** |
|  | **Smoker** | 0.96 (0.65-1.41) | 0.91 (0.58-1.43) | -0.09 | **-1** |
| **Physical inactivity**^€^ | **Physically active** | Reference | - | - | **0** |
|  | **Physically inactive** | 1.48 (1.14-1.93) | 1.33 (1-1.76) | 0.28 | **3** |
|  | **Constant** |  |  | -2.64 |  |

^¥^Defined as body mass index ≥ 30 kg/m^2^.^1^

^#^Defined as those currently smoking tobacco daily.^1^

^€^Defined as <600 metabolic equivalent-minutes per week (i.e., <150 minutes per week of brisk walking or <75 minutes per week of running).^1^

**Table S4.** Multivariable logistic regression of risk factors for type 2 diabetes mellitus on the model-simulated sample of the Qatari population to formulate the *model-derived* risk score for the year 2012.

|  |  | **OR (95% CI)** | **aOR (95% CI)** | **β** | **Risk score*** |
| --- | --- | --- | --- | --- | --- |
| **Age group** | **15-19** | Reference | - | - | **0** |
|  | **20-24** | 2.43 (1.51-3.91) | 1.96 (1.2-3.18) | 0.67 | **7** |
|  | **25-29** | 4.13 (2.61-6.55) | 2.55 (1.59-4.09) | 0.94 | **9** |
|  | **30-34** | 4.74 (2.98-7.52) | 2.73 (1.69-4.39) | 1.00 | **10** |
|  | **35-39** | 6.35 (4.03-10.01) | 3.36 (2.1-5.37) | 1.21 | **12** |
|  | **40-44** | 9.08 (5.75-14.32) | 4.61 (2.88-7.37) | 1.53 | **15** |
|  | **45-49** | 16.13 (10.24-25.42) | 8.73 (5.45-13.96) | 2.17 | **22** |
|  | **50-54** | 18.15 (11.5-28.65) | 9.54 (5.95-15.28) | 2.26 | **23** |
|  | **55-59** | 18.15 (11.37-28.96) | 11.03 (6.8-17.87) | 2.4 | **24** |
|  | **60-64** | 16.06 (9.82-26.26) | 9.48 (5.7-15.78) | 2.25 | **22** |
|  | **65-69** | 14.13 (8.46-23.61) | 9.29 (5.44-15.84) | 2.23 | **22** |
|  | **70-74** | 12.87 (7.51-22.05) | 9.93 (5.69-17.33) | 2.3 | **23** |
|  | **75-79** | 11.27 (6.32-20.1) | 9.24 (5.06-16.85) | 2.22 | **22** |
| **Sex** | **Women** | Reference | - | - | **0** |
|  | **Men** | 1.19 (1.03-1.38) | 1.43 (1.2-1.7) | 0.36 | **4** |
| **Obesity**^¥^ | **Non-obese** | Reference | - | - | **0** |
|  | **Obese** | 5.09 (4.334-5.97) | 4.28 (3.6-5.1) | 1.45 | **15** |
| **Smoking**^#^ | **Non-smoker** | Reference | - | - | **0** |
|  | **Smoker** | 1.37 (1.13-1.65) | 1.08 (0.86-1.36) | 0.08 | **1** |
| **Physical inactivity**^€^ | **Physically active** | Reference | - | - | **0** |
|  | **Physically inactive** | 2.06 (1.78-2.39) | 1.66 (1.4-1.96) | 0.51 | **5** |
|  | **Constant** |  |  | -4.22 |  |

^¥^Defined as body mass index ≥ 30 kg/m^2^.^1^

^#^Defined as those currently smoking tobacco daily.^1^

^€^Defined as <600 metabolic equivalent-minutes per week (i.e., <150 minutes per week of brisk walking or <75 minutes per week of running).^1^

**Table S5.** Validation of the model-derived Qatari diabetes risk score. Performance of the *model-derived* 2012 diabetes risk score as applied to the (empirical) 2012 STEPwise survey sample^1^ compared to the performance of the *data-derived* diabetes risk score as applied to this same sample.

| Risk score | AUC^#^ (95% CI^$^) | Sensitivity (%; 95% CI) | Specificity (%; 95% CI) | PPV^&^  (%; 95% CI) | NPV^*^  (%; 95% CI) | Risk score cut-off | Proportion needed testing (%; 95% CI) |
| --- | --- | --- | --- | --- | --- | --- | --- |
| 2012 STEPwise survey score | 0.70  (0.68-0.73) | 57.8  (51.9-63.5) | 72.1  (69.5-74.6) | 32.6  (28.6-36.9) | 88.0  (85.8-89.9) | 12.5 | 33.6  (31.2-36.0) |
| 2012 Qatari diabetes risk score | 0.69  (0.66-0.72) | 62.9  (57.1-68.4) | 64.0  (61.2-66.6) | 28.9  (25.4-32.7) | 88.1  (85.7-90.1) | 27.5 | 41.1  (38.6-43.7) |

**^#^**AUC: Area under the curve; ^$^CI: Confidence interval; ^&^PPV: Positive predictive value; ^*^NPV: Negative predictive value.

**References**

1 Supreme Council of Health. Qatar STEPwise report 2012: Chronic disease risk factor surveillance (available at: <http://www.who.int/chp/steps/qatar/en/>). (2013).
